# The effects of parental mindfulness on preschoolers’ prosocial behavior: the chain mediating model of marital quality, authoritative parenting, and the sex difference

**DOI:** 10.3389/fpsyg.2026.1703808

**Published:** 2026-02-12

**Authors:** Mengmeng Zhang, Lei Qiao, Yijing Zhang, Li Li, Shangning Zhao, Tosin Yinka Akintunde, Xiantong Yang

**Affiliations:** 1 School of Education, Fujian Normal University, Fuzhou, China; 2 Academic Affairs Office, Pu’er University, Pu’er, China; 3 College of Education, University of Maryland, College Park, MD, United States; 4 Faculty of Teacher Education, Pu’er University, Pu’er, China; 5 School of Education, Ningxia Preschool Education College, Ningxia, China; 6 Faculty of Nursing, University of Alberta, Edmonto, AB, Canada; 7 School of Psychology, Fujian Normal University, Fuzhou, China

**Keywords:** authoritative parenting, marital quality, mindfulness, preschool children, prosocial behavior

## Abstract

Family system factors have been consistently linked to children’s prosocial behavior. However, the mechanisms underlying these associations remain unclear. Drawing on family system theory, attachment theory, and social learning theory, the present study examined the associations between parental mindfulness and preschoolers’ prosocial behavior, as well as the mediating roles of marital quality and authoritative parenting, using data from 754 Chinese parents of preschool children. Results indicated that parental mindfulness was positively associated with marital quality, authoritative parenting, and preschoolers’ prosocial behavior. In addition, parental mindfulness was indirectly associated with prosocial behavior through marital quality and authoritative parenting, both independently and sequentially. Multigroup comparison revealed all paths were significant in the boy group. However, in the girl group, only the independent mediating effect of authoritative parenting and the chain mediation effect of marital quality and authoritative parenting were significant. Notably, the effect size was larger in the girl group for both the independent mediating path of authoritative parenting and the chain mediation path of marital quality and authoritative parenting. Overall, these findings clarify the relational and parenting processes linking parental mindfulness with preschoolers’ prosocial behavior.

## Introduction

Prosocial behavior refers to self-motivated behaviors that are beneficial to others, including helping, sharing, comforting, and guiding (Eisenberg et al., 1998). The development of prosocial behavior in childhood represents a core component of socialization and personality development and has been linked to long-term developmental trajectories (Lan and Wang, 2022). Previous research has consistently shown that prosocial behaviors are associated with a range of positive outcomes, including peer acceptance (Guo et al., 2018), social adjustment (Crick, 1996), and academic achievement (Collie et al., 2018). Recent studies have increasingly focused on sociocultural, psycho-cognitive, and other multidimensional factors associated with children’s prosocial behavior. From multiple perspectives, researchers have clarified the mechanisms and individual differences in the occurrence of children’s prosocial behavior by examining “mindfulness-prosocial behavior” (Siu et al., 2016), “marital quality-prosocial behavior”(Zhang et al., 2022), and “parenting-prosocial behavior” (Ziv and Arbel, 2021). Few empirical studies have integrated multiple factors within a framework, as most prior research has examined these elements in isolation. The family system theory states that subsystems within a family are interdependent, and parents’ emotions and behaviors in the family spill over into the parenting subsystem, directly or indirectly influencing children’s prosocial behavior (Cox and Paley, 1997). Thus, the present study examines the associations among parental mindfulness, marital quality, authoritative parenting, and children’s prosocial behavior.

### Parental mindfulness and prosocial behavior

Parental mindfulness has emerged as a crucial factors of preschoolers’ prosocial behavior (Geurtzen et al., 2015; Siu et al., 2016; Chen et al., 2022). Parental mindfulness was defined as paying attention to the present moment and expressing acceptance, kindness, and compassion during the parenting process (Kabat-Zinn, 2003; Hertz et al., 2015). Research has shown that mindful parenting is associated with reductions in children’s emotional symptoms, conduct problems, and increased prosocial behavior (Siu et al., 2016; Geurtzen et al., 2015). Children may internalize mindful interaction patterns, such as parental acceptance and understanding, through observation and imitation, which may be reflected in their prosocial behaviors during peer interactions (Chen et al., 2022). Therefore, parental mindfulness may be closely associated with preschoolers’ prosocial behavior.

### Parental mindfulness, marital quality, and prosocial behavior

Parental mindfulness may be closely associated with marital quality. Marital quality refers to married couples’ subjective evaluations of satisfaction and wellbeing within their marital (Glenn, 1990). Prior research has shown that higher levels of mindfulness are associated with greater intimacy between couples and lower levels of family conflict (Quinn-Nilas, 2020). Mindful individuals tend to exhibit greater awareness and emotional regulation in interpersonal contexts, which may be linked to reduced impulsive reactions and less critical or indifferent communication patterns, and in turn, to stronger emotional connectedness between partners (Cox et al., 2020). Similarly, studies on mindfulness-based intervention have demonstrated that individual with high levels of mindfulness are associated with healthier marital functioning (Faircloth and Cummings, 2008).

Moreover, parents’ marital quality has been consistently associated with the children’s prosocial behavior. Family systems theory posits that family subsystems are interdependent, such that parents’ interactions within the marital subsystem are linked to children’s behavioral functioning through broader family dynamics (Weeland et al., 2021). Research suggests that higher marital quality is associated with more harmonious family interactions and more supportive home environments, which are, in turn, related to children’s social and behavioral adjustment (Mark and Pike, 2017). In contrast, lower marital quality has been linked to greater marital conflict and emotionally withdrawn interaction patterns, which are associated with increased risks of children’s emotional and behavioral difficulties, including internalizing problems such as anxiety and depression (Harold and Sellers, 2018). In summary, we could reasonably propose that parental mindfulness was a facilitator of marital quality, which would in turn improve preschoolers’ prosocial behavior.

### Parental mindfulness, authoritative parenting, and prosocial behavior

Authoritative parenting may represent an important relational pathway linking parental mindfulness with preschoolers’ prosocial behavior. Authoritative parenting is characterized by high levels of parental responsiveness and warmth and reflects parenting attitudes that emphasize coordination, support, and acceptance of children’s needs and demands (Chen et al., 1997; Hosokawa and Katsura, 2017). Based on the mindful parenting theoretical model (Duncan et al., 2009), it emphasizes that parents with high levels of mindfulness are more likely to adopt positive parenting (e.g., consistent discipline, monitoring, positive interaction, warmth expression) and exhibit lower levels of harsh parenting (Duncan et al., 2015; Gouveia et al., 2016; Parent et al., 2016).

Additionally, authoritative parenting has been associated with children’s prosocial behavior. Social learning theory indicates children acquire behaviors by observing and imitating others (O’Connor et al., 2013). Children need authoritative parents to model positive behavior by accepting, caring for, and supporting them in the early years of their lives. Children develop prosocial behaviors when they are taught to treat others with warmth and compassion (Carlo et al., 2018). Overall, we could reasonably hypothesize that parental mindfulness may be positively associated with authoritative parenting, which in turn may improve preschoolers’ prosocial behavior.

### Marital quality and authoritative parenting

Children’s prosocial behavior may be associated with marital quality and authoritative parenting. The marital subsystem is central to family life and has a direct impact on the parent-child subsystem. According to the spillover hypothesis, positive emotions generated within a high-quality marriage enable parents to be more sensitive to the needs of their children. Such sensitivity has been linked to higher levels of understanding, encouragement, and warmth in parenting practices, which are, in turn, associated with children’s prosocial behavior (Pinquart, 2017). Conversely, parents who are chronically exposed to marital conflict may undermine effective parenting because they tend to absorb a great deal of emotion from the couple’s destructive behaviors and carry this negative emotion into the parent-child subsystem, resulting in decreased parental warmth and responsiveness toward their children. A meta-analysis of marital quality and parenting style also confirmed a significant association between negative parenting and parental conflict (Krishnakumar and Buehler, 2000).

Additionally, through positive dialogue and communication with their partners, parents with high levels of mindfulness can reduce their impulsive behaviors and improve the quality of their marriages by using mindfulness techniques. The relationship between high-quality marriage and authoritative parenting creates positive emotional feelings in parents, which in turn provides a template for children to learn how to care for, understand, and comfort others (Wang et al., 2015). Therefore, based on the above review, we could plausibly predict that the influence of parental mindfulness on preschoolers’ prosocial behavior may be mediated by marital quality and authoritative parenting.

### Children’s sex differences

Given sex-differentiated socialization experiences, it is necessary to verify whether there are differences in the hypothesized model’s pathway relationships between boys and girls. Prior research suggests that parents often engage in sex-differentiated parenting practices, with girls more likely to receive emotional support and relational guidance than boys (Shek, 2008). Within the Chinese cultural context, traditional sex role expectations emphasize emotional attunement and interpersonal sensitivity more strongly for girls than for boys (Lan and Wang, 2022). In addition, social expectations regarding masculinity and femininity may shape parenting styles, such that boys are more frequently socialized toward independence and assertiveness under stricter discipline, whereas girls are more often encouraged to be caring, empathetic, and emotionally expressive within more supportive and gentle parenting contexts (Bosak et al., 2008). Previous research has also confirmed that parents show more positive attention and emotional interaction with their daughters (Volling et al., 2002).

Gender role theory further suggests that girls may be more attentive to emotional cues within their interpersonal environment and more responsive to variations in family relational climates (Hu and Cheung, 2021). Given that the marital relationship constitutes a central relational context within the family system, variations in marital quality may be more strongly associated with girls’ emotional and behavioral functioning, including prosocial behavior (Lan and Wang, 2022). In light of these theoretical and empirical considerations, the present study examines potential sex differences in the proposed pathways. Specifically, we hypothesize that the associations among parental mindfulness, marital quality, authoritative parenting, and preschoolers’ prosocial behavior may be more pronounced among girls than among boys.

### The current study

Although prior research has established the importance of mindfulness, most studies have focused on its effects at the individual level. There remains a need to examine the role of mindfulness within the family microecosystem, particularly its associations with marital quality, parenting practices, and children’s social development. Moreover, existing research has typically examined parental mindfulness, marital quality, or parenting style in isolation, with few studies exploring the mechanisms linking these factors with prosocial behavior. Moreover, it remains unclear whether these mechanisms differ by children’s sex.

**FIGURE 1:**
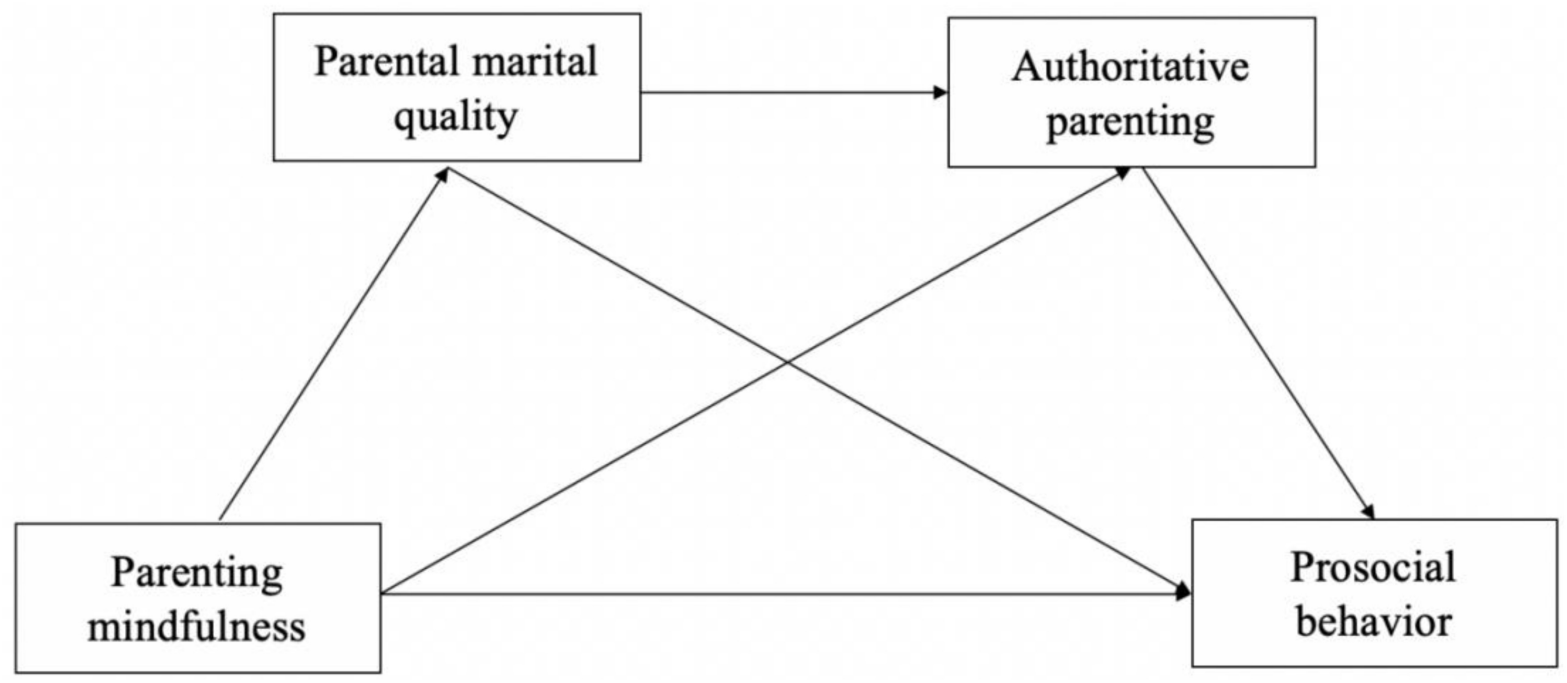
Conceptual framework of marital quality and authoritative parenting as mediators.

Based on the above literature, parental mindfulness appears to be positively associated with preschoolers’ prosocial behavior. In existing studies examining the potential mechanisms underlying this association, marital quality and authoritative parenting have been suggested as important family relational processes linking parental characteristics to children’s social development. Moreover, previous research indicates that these family processes may operate jointly within the family system, and that such associations may differ by children’s sex. As an integrated model, the present study was guided by the following hypotheses (see Figure 1).

*H1*: Parental mindfulness would be positively associated with preschoolers’ prosocial behavior.

*H2*: Marital quality would play a mediating role in the association between parental mindfulness and preschoolers’ prosocial behavior.

*H3*: Authoritative parenting would play a mediating role in the association between parental mindfulness and preschoolers’ prosocial behavior.

*H4*: Parental mindfulness would be indirectly associated with preschoolers’ prosocial behavior through the sequential mediation of marital quality and authoritative parenting.

*H5*: Sex differences would be observed in the hypothesized model.

## Materials and methods

### Participants

This study employed a cross-sectional design involving parents of children aged 3–7 years in the Ningxia Hui Autonomous Region, China, from September to October 2022. To ensure data quality, we initially contacted 20 kindergartens and obtained their approval. Principals then informed parents about the survey’s purpose. Participation was voluntary, and only parents with children aged between 3 and 7 years were invited to participate. Parents completed the questionnaire anonymously in a relaxed and distraction-free environment to encourage honest responses. The questionnaire was designed to require responses to all items, resulting in no missing data in the initial dataset. The initial sample included 820 participants. However, after applying attention check criteria, 36 participants were excluded, along with 30 participants due to excessively short response times. Consequently, the final valid sample comprised 754 participants with complete data.

Among these parents, 117 were fathers and 637 were mothers. The mean age of the fathers and mothers were 36.45 and 34.56, respectively. Regarding educational level, 75 (9.9%) fathers and 461 (61.1%) mothers reported to be post-secondary graduates/high school graduates, 40 (5.3%) fathers and 169 (22.4%) mothers were undergraduates, and 2 fathers (0.2%) and 7 (0.9%) mothers claimed to be postgraduates.

### Measures

#### Five facet mindfulness questionnaire

This study used the Chinese version of the Five Facet Mindfulness Questionnaire (FFMQ) revised by Deng et al. (2011), which was adapted from the original questionnaire developed by Baer et al. (2006). The FFMQ consists of five facets: Observing (e.g., “I am aware of the sensation of water as it flows over my body during a shower”), Describing (e.g., “I can easily put my beliefs, opinions, and expectations into words”), Non-judging (e.g., “I refrain from criticizing myself for the thoughts I’m having right now”), Non-reactivity to inner experience (e.g., “I can observe my thoughts and feelings without getting lost in them”), and Acting with awareness (e.g., “I find myself doing things with intention and attention”). The questionnaire contains 39 items and is scored on a 5-point scale ranging from 1 = “*completely disagree*” to 5 = “*completely agree.*” The internal consistency of the total scale in this study was 0.770, and the internal consistency of each subscale ranged from 0.678 to 0.863, indicating suitable reliability.

#### Marital quality index

This study adapted the Marital Quality Index (MQI) developed by Norton (1983) to assess marital quality. The MQI is a unidimensional scale consisting of six items (e.g., “My relationship with my partner is very stable”), with the first five items rated on a five-point scale ranging from 1 = “*strongly disagree*” to 5 = “*strongly agree*” and the sixth item rated on a 10-point scale ranging from 1 = “*very dissatisfied*” to 10 = “*very satisfied.*” Higher scores indicate better marital quality. To ensure comparability, all items were standardized (*z*-scores) before computing the total score. The internal consistency coefficient of the scale in this study was 0.86, indicating good reliability.

#### Parenting style questionnaire

The Parenting Style Questionnaire (PSQ) was used to assess authoritative parenting style (Wu et al., 2002). The authoritative parenting style consists of three subscales: warmth and acceptance (e.g., “I show understanding and comfort when my child is upset”), reasoning and induction (e.g., “I explain to my child the consequences of their behavior”), and democratic participation (e.g., “I allow my child to participate in setting family rules”). The PSQ contains 15 items rated on a five-point scale ranging from 1 = “*never*” to 5 = “*always*,” with higher scores indicating greater levels of authoritative parenting. The internal consistency coefficient of the PSQ in this study was 0.913, indicating good reliability.

#### The strengths and difficulties questionnaire

The Strengths and Difficulties Questionnaire (SDQ) was used to assess prosocial behavior in preschoolers (Goodman, 1997). The Strengths subscale consists of 5 items (e.g., “My child is willing to share things with others”), rated on a 5-point scale ranging from 1 (*completely disagree*) to 5 (*completely agree*). Higher scores indicate better prosocial behavior. The internal consistency coefficient of the SDQ in this study was 0.715, indicating acceptable reliability.

### Statistical analysis

First, descriptive statistics and Pearson’s correlations among the main variables were computed using SPSS version 23.0. Next, controlling for sex and age, structural equation modeling (SEM) was conducted using Mplus version 7.1 to examine the mediating effects of marital quality and authoritative parenting on the relationship between parental mindfulness and children’s prosocial behavior. SEM analyses employed the robust maximum likelihood estimator (MLR) to account for potential deviations from normality. Prior to SEM, data normality was evaluated by examining skewness and kurtosis of key variables, which were found to be within acceptable ranges. Finally, the multi-group (boys vs. girls) structural model comparison was used to test whether the associations in the chain mediation model varied with children’s sex. Besides, to determine the degree of fit between the survey data and the hypothesis model, the following fitting statistical scores were calculated: The Chi squared goodness of fit test (χ^2^) the comparative fit index (CFI), the Tucker-Lewis index (TLI), the root means square error of approximation (RMSEA), and the standardized root mean square residual (SRMR). We used the cutoff criteria recommended by Hu and Bentler (1999) to evaluate the data fit: CFI ≥ 0.90, TLI ≥ 0.90, RMSEA < 0.08, and SRMR < 0.1.

## Research results

### Common method variance

Since the data were gathered through parents’ self-reports, the results may be subject to common method variance. Therefore, Harman’s single-factor test was conducted to assess potential systematic measurement error (Harman, 1960). The results indicated that 11 factors with eigenvalues greater than 1 were extracted, and the first factor accounted for 19.12% of the total variance, which is below the 40% threshold suggested by Podsakoff et al. (2003).

### Descriptive statistics and correlation analysis

Table 1 shows the means, standard deviations, and bivariate correlations for main variables. Correlation analysis indicated that parental mindfulness was significantly positively correlated with marital quality, authoritative parenting, and children’s prosocial behavior (*r* ranged from 0.248 to 0.523). Marital quality was significantly positively correlated with authoritative parenting (*r* = 0.277) and children’s prosocial behavior (*r* = 0.225). Authoritative parenting style was significantly positively associated with children’s prosocial behavior (*r* = 0.500).

**TABLE 1:** Descriptive statistics and correlation coefficients of key variables.

Variables	*M* ± SD	1	2	3	4
1. Parental mindfulness	3.167 ± 0.305	1	1	1	1
2. Marital quality	29.514 ± 4.999	0.248**			
3. Authoritative parenting	3.982 ± 0.572	0.523***	0.277**		
4. Prosocial behavior	3.933 ± 0.558	0.346***	0.225**	0.500***	

**p < 0.01;

***p < 0.001.

### The analyses of chain mediating effects

The direct effect of parental mindfulness on preschoolers’ prosocial behavior was examined using Mplus 7.1. Controlling for demographic variables such as parental age and sex, results indicated that parental mindfulness was significantly positively associated with children’s prosocial behavior (standardized path coefficient,β = 0.346, *p* < 0.001). The model demonstrated good fit indices: χ^2^ = 2.470, *df* = 1, CFI = 0.985, TLI = 0.955, RMSEA = 0.044, SRMR = 0.018.

**FIGURE 2:**
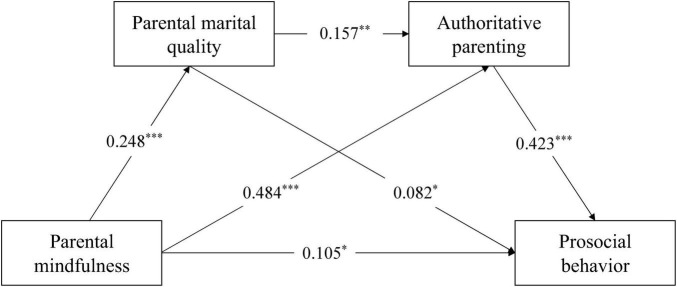
The chain mediation model. *p < 0.05; **p < 0.01; ***p < 0.001.

Second, a chain mediation model was constructed to examine the associations among parental mindfulness, marital quality, authoritative parenting, and children’s prosocial behavior. The results indicated a good model fit (χ^2^ = 3.991, *df* = 3, CFI = 0.998, TLI = 0.994, RMSEA = 0.021, SRMR = 0.018). As shown in Figure 2, all path coefficients were statistically significant. Specifically, parental mindfulness was positively associated with marital quality (β = 0.248, *p* < 0.001) and authoritative parenting (β = 0.484, *p* < 0.001). Marital quality was significantly associated with authoritative parenting (β = 0.157, *p* < 0.01) and children’s prosocial behavior (β = 0.082, *p* < 0.05). Authoritative parenting was positively associated with children’s prosocial behavior (β = 0.423, *p* < 0.001).

Next, a bootstrap procedure with 5,000 resamples was employed to test the chain mediation effect. The 95% confidence interval for the chain mediating path from parental mindfulness to children’s prosocial behavior was [0.001, 0.009]. Since the interval did not include zero, the chain mediation effect was considered significant. The estimates and effect sizes for each mediation path are presented in Table 2. The direct effect of parental mindfulness on children’s prosocial behavior was significant [β = 0.105, 95% CI (0.032, 0.180)]. The total mediating effect was also significant [β = 0.241, 95% CI (0.171, 0.328)], accounting for 69.65% of the total effect. Namely, three indirect paths were identified: parental mindfulness → marital quality → preschoolers’ prosocial behavior, parental mindfulness → authoritative parenting → preschoolers’ prosocial behavior, and parental mindfulness → marital quality → authoritative parenting → preschoolers’ prosocial behavior, accounting for 5.78, 59.25, and 4.62% of the effect sizes, respectively.

**TABLE 2:** SEM path coefficients.

Effect	Pathway	Effect size	95% CI	Relative mediation effect
Indirect effect	Parental mindfulness → children prosocial behavior	0.105	[0.032, 0.180]	30.35%
Mediating effect	Indirect path 1: parental mindfulness → marital quality → children prosocial behavior	0.02	[0.062, 0.128]	5.78%
	Indirect path 2: parental mindfulness → authoritative parenting → children prosocial behavior	0.205	[0.008, 0.084]	59.25%
	Indirect path 3: parental mindfulness → marital quality → authoritative parenting → children prosocial behavior	0.016	[0.001, 0.009]	4.62%
	–	0.241	[0.171, 0.328]	69.65%
Total effect	–	0.346		100%
Comparison 1 (path 1 and path 2)	–	0.047	[−0.002, 0.099]	–
Comparison 2 (path 1 and path 3	–	0.089	[0.059, 0.124]	–
Comparison 3 (path 2 and path 3)	–	0.042	[0.008, 0.078]	–

Finally, pairwise comparison tests were conducted to examine differences among the indirect paths (see Table 2). For Comparison 1, the 95% bootstrap confidence interval for the difference between indirect path 1 and indirect path 2 was [−0.433, −0.267], which does not include zero, indicating a significant difference between the two paths. For Comparisons 2 and 3, the 95% bootstrap confidence intervals were [0.059, 0.124] and [0.008, 0.078], respectively, both excluding zero, also indicating significant differences between path 1 and path 2, and between path 2 and path 3.

### Multigroup comparison

**FIGURE 3:**
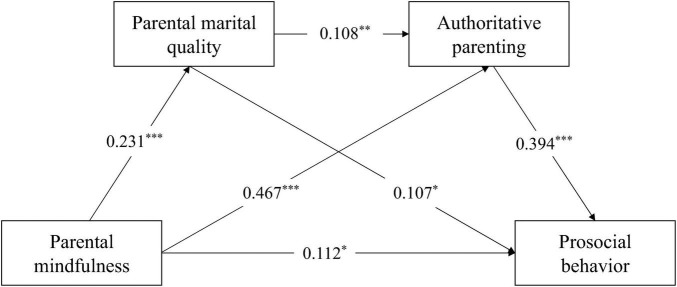
The chain mediation model (boy group). *p < 0.05; **p < 0.01; ***p < 0.001.

**FIGURE 4:**
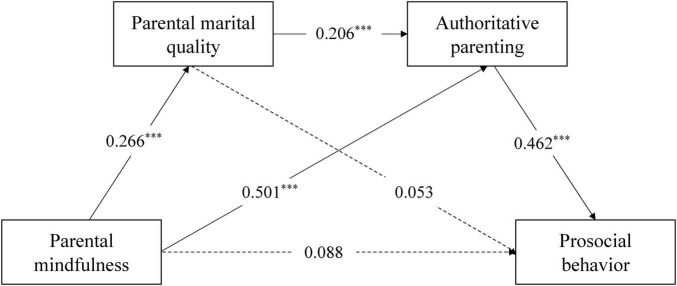
The chain mediation model (girl group). ***p < 0.001.

To investigate whether the chain mediation model exhibits consistency across children sex, a measurement invariance test was first conducted. First, a test of configural invariance (M1) indicated that the chain mediating effect for sex was measurement invariant. Second, since the M2 model was nested within the M1 model, a loading equivalence model (M2) test was conducted using a nested model comparison approach (Liu et al., 2007). The fit indices suggested that the chain mediating effect exhibits sex differences in children (Δχ^2^ = 24.29, Δ*df* = 26, *p* < 0.001). Then, we compared the chain mediation models for different sex (see Figures 3, 4). The results showed that: (1) The direct effect in the boy group was significant, and both the independent mediating effects and chain mediation effects of marital quality and authoritative parenting were significant. (2) The direct effect in the girl group was not significant, but the independent mediating effect of authoritative parenting and the chain mediation effect of marital quality and authoritative parenting were significant. (3) In the independent mediating path of authoritative parenting, the effect size in the girl group (0.231) was larger than that in the boy group (0.184), indicating that the indirect association between parental mindfulness and prosocial behavior via authoritative parenting was stronger for daughters than for sons. Similarly, in the chain mediation path of marital quality and authoritative parenting, the effect size in the girl group (0.025) was larger than that in the boy group (0.010), indicating a stronger indirect association linking parental mindfulness to prosocial behavior through marital quality and authoritative parenting in daughters than in sons.

## Discussion

The association between parental mindfulness and children’s prosocial behavior has acquired empirical support (Geurtzen et al., 2015; Siu et al., 2016; Chen et al., 2022). Nevertheless, the mediating mechanisms underlying the association remained unclear. Drawing on family systems, attachment, and social learning theories, this study proposed a chain mediation model to examine these pathways. Family systems theory highlights the spillover of marital quality into parenting practices, attachment theory emphasizes the role of authoritative parenting in fostering secure bonds that support prosocial development, and social learning theory focuses on children’s acquisition of prosocial behaviors through parental modeling. Consistent with this integrated framework, the results indicated that parental mindfulness was significantly and positively associated with preschoolers’ prosocial behavior, marital quality, and authoritative parenting. Moreover, parental mindfulness was indirectly associated with prosocial behavior through the indirect path of marital quality and authoritative parenting, as well as the chain mediating path between these two variables. Multigroup comparisons further revealed sex differences in the chain mediation model. These findings provide a comprehensive and constructive perspective, shedding light on how family system factors jointly relate to preschoolers’ prosocial behavior.

### The association between parental mindfulness and preschoolers’ prosocial behavior

Consistent with previous research (Siu et al., 2016), parental mindfulness was positively associated with preschoolers’ prosocial behavior. Hypothesis 1 was confirmed. From a socialization perspective, mindful parents tend to demonstrate greater awareness, emotional openness, and empathy in daily interactions, which may provide children with opportunities to observe and internalize prosocial behaviors (Chen et al., 2022). Through repeated parent-child interactions, children may generalize these observed behaviors to broader social contexts, such as peer relationships (Siu et al., 2016). In this sense, parental mindfulness appears to be linked to children’s prosocial behavior by shaping the emotional and interpersonal climate in which children develop.

### The mediating role of marital quality

The results identified the mediating role of marital quality in the association between parental mindfulness and children’s prosocial behavior. Hypothesis 2 was confirmed. Specific to the first stage of the mediation link, parental mindfulness was positively associated with marital quality, in line with previous research (Faircloth and Cummings, 2008). Couples with high mindfulness thoughts are more likely to practice mindfulness techniques that reduce impulsive behavior and communication of criticism and indifference, which fosters an emotional connection between them (Cox et al., 2020). Moreover, the results also manifested that marital quality was significantly associated with prosocial behavior. From a family systems perspective (Weeland et al., 2021), the quality of the marriage affects the dynamics of the home, how family members interact, and how children behave (Mark and Pike, 2017). A healthy marriage fosters a happy home environment, gives children more encouraging feedback and warm support, and encourages prosocial behavior in children (Wang et al., 2022). Taken together, our findings showed that parental mindfulness was positively associated with marital quality, which in turn was related to preschoolers’ prosocial behavior.

### The mediating role of authoritative parenting

Parental mindfulness was positively associated with authoritative parenting, which in turn was related to preschoolers’ prosocial behavior, supporting Hypothesis 3. Parents with higher levels of mindfulness are better able to respond calmly and acceptingly to their children’s inappropriate behavior, while actively communicating to resolve conflicts (Williams and Wahler, 2010). These interaction patterns are consistent with the core characteristics of authoritative parenting. Furthermore, authoritative parenting was positively associated with increased prosocial behavior in preschoolers, in line with attachment theory (Bretherton, 1992). Authoritative parents tend to create a secure physical and social environment that fosters children’s emotional attachment, which in turn encourages positive interpersonal interactions and prosocial development (Raver, 2004). Similarly, Bandura’s social learning theory also suggests that children acquire social behaviors through observation and imitation (O’Connor et al., 2013). Parents with authoritative parenting provide role models of warm, accepting, and supportive behavior to their children, which facilitate their children’s ability to learn and exhibit prosocial behaviors in social interactions (Carlo et al., 2018). Overall, our findings showed that parental mindfulness was positively associated with authoritative parenting, which in turn was related to preschoolers’ prosocial behavior.

### Chain mediation of marital quality and authoritative parenting

Finally, parental mindfulness was indirectly associated with higher levels of prosocial behavior through its positive associations with marital quality and authoritative parenting, consistent with Hypothesis 4. The finding indicated that the process of parental mindfulness on preschoolers’ prosocial behavior was relatively complex. Specifically, mindful parents are better able to recognize, regulate, and manage their emotions in stressful or conflictual situations, which helps reduce impulsive behaviors between partners and supports the maintenance of healthy marital relationships (Cox et al., 2020). According to the spillover hypothesis (Pinquart, 2017), emotions generated within the marital relationship tend to permeate the parent-child system. Positive emotions arising from cooperative and constructive marriages motivate parents to engage in more affectionate and involved parenting. Such parenting, characterized by greater sensitivity, understanding, care, and supportive encouragement, fosters the development of children’s prosocial behavior (Zhang et al., 2022). Overall, this study extends the literature by providing empirical evidence that parental mindfulness is associated with preschoolers’ prosocial behavior through its links with marital quality and authoritative parenting.

### Comparative analysis of multiple groups for children’s sex

The association between parental mindfulness and preschoolers’ prosocial behavior differed by child sex, partially supporting Hypothesis 5. Both boys and girls demonstrated an independent mediating effect of authoritative parenting, as well as a chain mediating effect involving marital quality and authoritative parenting. However, these effects were stronger in girls than in boys. Notably, only boys group showed an independent mediating effect of marital quality between parental mindfulness and prosocial behavior. These findings suggest that boys may be more susceptible to the interpersonal environment.

There are some potential underlying reasons for this phenomenon. Within the Chinese cultural context, girls tend to display higher emotional dependence, which is related to traditional gender norms (Lan and Wang, 2022). Parents often adopt sex-differentiated parenting approaches, providing relatively more psychological support to girls than boys (Shek, 2008), which may account for the stronger association between authoritative parenting and prosocial behavior in girls. Conversely, boys appear to be more sensitive to differences in interpersonal relationships (Leaper et al., 1989; Wang et al., 2022). In families where parents maintain a harmonious marital relationship, children’s cognitive empathy and perspective-taking abilities are likely to be more developed, which corresponds with higher levels of prosocial behavior modeled by parents (Bernier et al., 2014). Additionally, based on the modeling and compensation hypotheses, an intimate and harmonious relationship between spouses can influence boys to model their parents’ relationship, learn appropriate emotional and behavioral processing, and become more prosocial (Wang et al., 2022). Hence, marital quality may have a greater association with boys’ prosocial behavior than on girls. This phenomenon needs to be explored and confirmed through further research.

## Limitations and implications

Despite the strengths and contributions of the present study, several limitations should be considered when interpreting the findings. First, cross-sectional research could not establish causal linkages across models, longitudinal and experimental investigations are needed to confirm these associations. Second, all variables were solely parent-reported, raising concerns about social desirability and subjective bias. To enhance the reliability and validity of the findings, future studies should employ multi-informant and multi-method approaches, including behavioral and physiological measures of mindfulness, partner reports and observational coding of marital quality, and a combination of observations and reports from others to assess parent-child dynamics and prosocial behavior. Third, the sample consisted predominantly of mothers (637 mothers vs. 117 fathers). Although parental sex was controlled in the analyses, this imbalance may limit the generalizability of the findings, particularly regarding paternal mindfulness and parenting behaviors. As a result, the findings may primarily reflect maternal experiences, and caution is warranted when generalizing them to fathers. Future studies should recruit more balanced samples to better capture potential gender differences. Fourth, although Harman’s single-factor test suggested that common method variance may not be severe, it cannot fully eliminate concerns regarding common method bias. The use of a single informant and self-report questionnaires may have contributed to inflated associations among the study variables. Accordingly, the findings should be interpreted with caution.

Despite these limitations, this study also has some implications. Theoretically, it may be beneficial to investigate the associations between parental mindfulness and preschoolers’ prosocial behavior, as this could provide a better understanding of the factors associated with preschoolers’ prosocial behavior and contribute to theoretical bases related to external environmental resources and individual cognition. From an educational decision-maker’s perspective, this study highlights the importance of parental mindfulness in relation to children’s prosocial behavior, alongside aspects such as authoritative parenting and marital quality.

In regard to targeted interventions to enhance preschoolers’ prosocial behavior, consideration of family system factors is important. Firstly, education departments in China have promoted mindful parenting interventions aimed at supporting parents’ emotional states. Mindfulness therapy has been associated with alleviating anxiety, improving positive emotional experiences, and fostering a calm, harmonious inner state. It is also linked with the healthy functioning of family subsystems, maintaining marital relationships, and children’s prosocial behaviors (Chen et al., 2022). Secondly, a harmonious family atmosphere is considered vital for children’s physical and mental development (Faircloth and Cummings, 2008). Among couples, effective communication and mutual respect are viewed as positive behavioral models related to children’s social development. Thirdly, parenting styles are connected with children’s psychological characteristics in socialization behaviors. Kindergartens may offer tailored parenting advice through various teaching methods, such as special lectures, teaching demonstrations, and parenting classes, which support authoritative parenting. Finally, parents are encouraged to acquire scientific parenting knowledge, reconsider traditional views, recognize sex differences in children’s social development, and provide comprehensive sex education tailored to their children’s individual characteristics.

## Conclusion

This study explored the mechanisms underlying the associations between parental mindfulness and preschoolers’ prosocial behavior. Parental mindfulness was examined as a factor potentially influencing family interactions and the overall family environment. The results suggested that parental mindfulness is positively associated with children’s prosocial behavior, with marital quality and authoritative parenting serving as separate indirect pathways. Additionally, parental mindfulness may relate to children’s prosocial behavior through a chain mediation effect involving both marital quality and authoritative parenting. These findings provide a theoretical foundation for improving children’s prosocial behavior as well as some practical guidance for parents and kindergarten administrators in developing effective interventions for promoting children’s prosocial behavior.

## Data Availability

The raw data supporting the conclusions of this article will be made available by the authors, without undue reservation.
